# Hospital admissions for skin and soft tissue infections in a population with endemic scabies: A prospective study in Fiji, 2018–2019

**DOI:** 10.1371/journal.pntd.0008887

**Published:** 2020-12-09

**Authors:** Li Jun Thean, Adam Jenney, Daniel Engelman, Lucia Romani, Handan Wand, Jyotishna Mudaliar, Jessica Paka, Tuliana Cua, Sera Taole, Aalisha Sahukhan, Mike Kama, Meciusela Tuicakau, Joseph Kado, Natalie Carvalho, Margot Whitfeld, John Kaldor, Andrew C. Steer

**Affiliations:** 1 Tropical Diseases Group, Murdoch Children’s Research Institute, Melbourne, Victoria, Australia; 2 Department of Paediatrics, University of Melbourne, Melbourne, Victoria, Australia; 3 College of Medicine, Nursing and Health Sciences, Fiji National University, Suva, Fiji; 4 Melbourne Children’s Global Health, Melbourne Children’s Campus, The Royal Children’s Hospital, Melbourne, Victoria, Australia; 5 Kirby Institute, University of New South Wales, Sydney, New South Wales, Australia; 6 Ministry of Health and Medical Services, Suva, Fiji; 7 Wesfarmers Centre for Vaccines and Infectious Diseases, Telethon Kids Institute, Perth, Western Australia, Australia; 8 School of Population and Global Health, University of Melbourne, Melbourne, Victoria, Australia; 9 Department of Dermatology, St. Vincent’s Hospital, Sydney, New South Wales, Australia; 10 School of Medicine, University of New South Wales, Sydney, New South Wales, Australia; Federal University of Ceará, Fortaleza, Brazil, BRAZIL

## Abstract

Scabies is an important predisposing factor for impetigo but its role in more serious skin and soft tissue infections (SSTIs) is not well understood. Information is limited on incidence of SSTIs in the presence of endemic scabies. We conducted a prospective study of hospital admissions for SSTIs in the Northern Division of Fiji (population: 131,914). Prospective surveillance for admissions with impetigo, abscess, cellulitis, wound infection, pyomyositis, necrotizing fasciitis, infected scabies, and crusted scabies was conducted at the Division’s referral hospital between 2018 to 2019. Information was collected on demographic characteristics, clinical features, microbiology, treatment and outcomes. Over the study period, 788 SSTI admissions were recorded corresponding to a population incidence 647 per 100,000 person-years (95%CI 571–660). Incidence was highest at the extremes of age with peak incidence in children aged <5 years (908 per 100,000) and those aged ≥65 years (1127 per 100,000). Incidence was 1.7 times higher among the Indigenous Fijian population (753 per 100,000) compared to other ethnicities (442 per 100,000). Overall case fatality rate was 3.3%, and 10.8% for those aged ≥65 years. Scabies was diagnosed concurrently in 7.6% of all patients and in 24.6% of admitted children <5 years. There is a very high burden of hospital admissions for SSTIs in Fiji compared to high-income settings especially among the youngest, oldest and indigenous population which is concordant with scabies and impetigo distribution in this population. Our findings highlight the need for strategies to reduce the burden of SSTIs in Fiji and similar settings.

## Author summary

Scabies is causally linked to impetigo in endemic populations. In turn, impetigo can progress to more serious skin and soft tissue infections (SSTI). However, there are few data on the epidemiology of SSTIs in settings where scabies is endemic. We conducted a prospective study of the incidence of hospitalizations for SSTIs in 2018 and 2019 at the referral centre for the Northern Division of Fiji (population of 131,914) where community scabies prevalence is very high. We measured the incidence of admissions for abscesses, cellulitis, impetigo, wound infections, pyomyositis, necrotizing fasciitis and crusted scabies. We observed a high incidence of SSTI admissions (647 cases per 100,000 person-years) with high associated morbidity and case fatality in this population. We found a very high incidence in young children, the elderly and iTaukei (Indigenous Fijian) population which is concordant with the distribution of scabies and impetigo in the community. These findings highlight SSTIs as an important public health concern and provide further impetus to advance research into strategies that will alleviate this burden.

## Introduction

Scabies is a pruritic skin disease caused by the mite *Sarcoptes scabiei* var. *hominis* with global prevalence estimated at 200 million in 2015. [1]Available studies have found high levels of prevalence in diverse, resource-limited settings including Pacific islands countries, parts of Latin America and some remote Aboriginal communities in Australia.[1] Scabies has a well described association with impetigo,[2–4] as breaches in skin from scratching facilitate acquisition of secondary bacterial infection, predominantly caused by *Staphylococcus aureus* and Group A *Streptococcus* (GAS).[5] A 2007 national survey in Fiji reported impetigo prevalence at 19.6% across all ages, and 34.2% in children aged 5 to 9 years.[2] Impetigo is understood to be a pathway by which more severe skin and soft tissue infections (SSTIs) and systemic infection can arise however the frequency of this progression is not well defined.

Thus, while skin and soft tissue infections are a key potential complication of scabies, little is known about their epidemiology, severity and outcomes in scabies endemic settings. According to a 2017 Global Burden of Disease Study update, a high proportion of the burden of skin and subcutaneous disease is caused by cellulitis and pyoderma, especially in Oceania and Africa, suggesting there is considerable morbidity and mortality from SSTIs in these settings.[6] The Fiji Ministry of Health and Medical Services 2016 Health Status Report stated that SSTIs were the fifth most common cause of mortality nationally.[7]

Clinical presentations of SSTIs range from superficial abscesses and cellulitis to life-threatening necrotizing fasciitis (also known as necrotizing soft tissue infections). In most populations where data are available, *S*. *aureus* and GAS are the most frequent causative pathogens, although attributable fractions vary by setting.[8–10] While most cases can be satisfactorily treated in primary care settings with standard antibiotics, a proportion require hospital admission.[11,12]

The incidence of SSTI admissions in high income countries such as the United States was reported as 219 per 100,000 person-years between 2005–2010. [12] A similar rate was found in non-Indigenous Australians at 290 per 100,000. However, this was substantially higher in the Indigenous population at 1,890 per 100,000,[13] suggesting an important role of environmental and socio-economic factors that may also be relevant in Pacific countries.[5] In a retrospective study of SSTI admissions to a public hospital in New Zealand, the incidence among children aged <14 years was 1352 per 100,000 among Pacific Islanders and 886 per 100,000 among Maori compared 229 per 100,000 among people of other ethnicities between 1990–2007.[14] Little is known about the incidence of SSTI admissions in low-middle-income or scabies endemic settings.

To better understand the occurrence of SSTIs in a setting with endemic scabies, we conducted a prospective study of admissions for SSTIs in the Northern Division of Fiji. This study was done as the baseline year of a before-after trial of ivermectin-based mass drug administration (MDA) for the control of scabies in this region (Trial ID: ACTRN12618000461291).

## Methods

### Ethics statement

Ethical approval was obtained from the Fiji National Health Research Ethics Review Committee (reference number: 2018.38.NOR.) and the Royal Children’s Hospital Human Research Ethics Committee in Melbourne, Australia (reference number: 38020). Written informed consent was obtained from all participants or from their parent or legal guardian if they were aged less than 18 years. If a potential participant was assessed to lack the capacity to provide properly informed consent, we sought consent from their primary carer or guardian. If consent was not obtained for medical records review, the case was retained for incidence and mortality calculations.

### Setting

Fiji is an archipelago nation in the South Pacific Ocean. Its population of approximately 885,000 people (2017), comprises two main ethnic groups, iTaukei or Indigenous Fijians (56.8%) and Fijians of Indian Descent (37.5%).[15,16] Fiji is ranked 98 out of 189 countries on the United Nations Development Programme Human Development Index 2019.[17]

**Fig 1:**
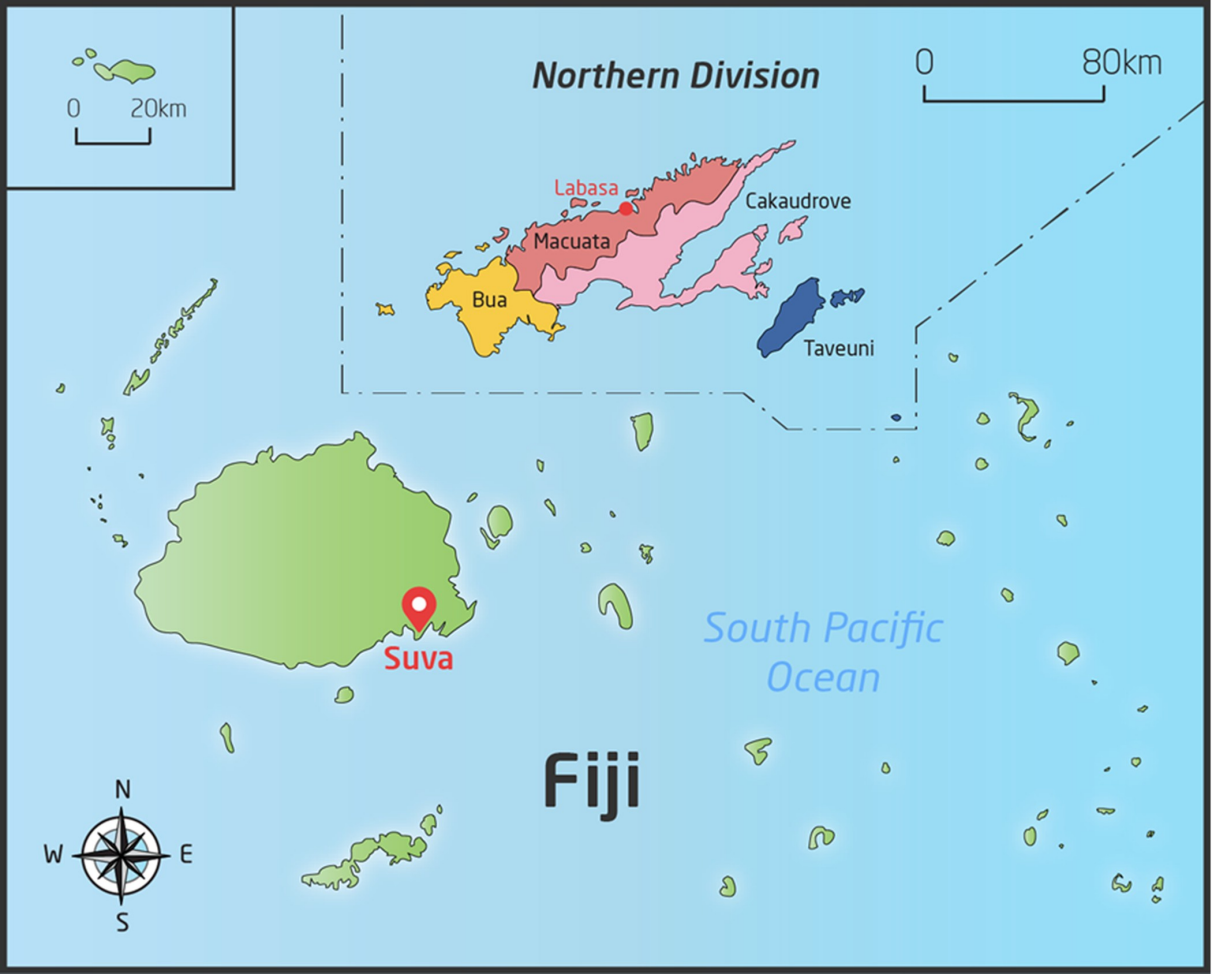
Map of the Northern Division of Fiji.

The Northern Division, with a population of 131,914 people in 2017 [15] is one of four administrative divisions of Fiji and is further divided into four subdivisions (Fig 1). The Northern Division was noted to have highest prevalence of scabies (28.5%) and impetigo (23.7%) by a national survey conducted in 2007.[2] Most of the population (70.6%) of the Northern Division live in rural areas. The study site was 195-bed Labasa Hospital, located in the divisional capital and the referral centre for the division. The other three subdivisions are serviced by smaller subdivisional hospitals.[18] Labasa Hospital is the only facility with specialist medical, surgical, paediatric, obstetric and intensive care unit (ICU) services, and a microbiology laboratory capable of processing specimens for bacterial culture.

### Surveillance and inclusion criteria

Following consultation with the Fiji Ministry of Health and Medical Services and administrative and ethical approvals, we established and implemented a protocol for prospective surveillance of SSTI admissions at Labasa Hospital over a 48-week period between July 16^th^ 2018 and June 30^th^ 2019, with a two week pause from 24^th^ December 2018 to 6^th^ January 2019.

Patients of all ages were recruited into the study. Potential study participants were identified through several processes to ensure comprehensive and accurate enrolment by two study nurses under the supervision of the study coordinator. First, ward-specific admission registries and case notes of new admissions in all inpatient wards of the hospital were reviewed daily, to identify possible cases. Patients discharged before contact could be made were followed up at home and offered enrolment. Second, a verbal check was conducted daily with the nurse in charge of each ward for any potential cases. Third, the hospital’s microbiology laboratory records were reviewed daily for skin swabs or operative soft tissue samples received and related back to the patient for potential enrolment.

Patients who met inclusion criteria were approached to give consent for review of their medical records. To be eligible, they had to have one of the following SSTIs: impetigo, abscess, cellulitis, wound infection (surgical and other), pyomyositis, necrotizing fasciitis, infected scabies, and crusted scabies. Diabetic foot infections were excluded. Eligibility was determined based on the working diagnoses of the treating clinicians as documented in the rounding books (a working ward book where diagnoses and plans for patients on each ward are documented daily), admission registries, case notes and on discharge, the hospital’s electronic record system Patient Information System (PATIS). Where necessary, clarification of diagnoses and treatment was sought from the treating clinicians. No study-specific assessments were implemented.

Detailed information from medical records regarding patient demographic, clinical and microbiological characteristics, management and outcomes were recorded after consent was obtained. Co-infestation with scabies as diagnosed by treating clinicians was noted. Surgical intervention was defined as a procedure performed in the operating theatre to treat the SSTI. Microbiological data were obtained from the microbiology laboratory records. Data were entered onto the REDCap Mobile App and securely stored in an online server hosted by the Murdoch Children’s Research Institute.[19,20]

### Microbiological methods

The microbiology laboratory at Labasa Hospital incubated blood culture bottles in an automated system (BacT/ALERT, bioMerieux, Craponne, France). Bacterial swabs were inoculated and incubated on solid media human/sheep blood agar, chocolate agar, Maconkey and cystine-lactose-electrolyte-deficient media. Identification of Gram-positive bacteria was performed through, Gram stain appearance and Oxoid Microbact (Thermo Fisher, Waltham, USA). *S*. *aureus* was identified through DNAse testing and GAS was identified through streptococcal latex agglutination test (Thermo Fisher) and bacitracin susceptibility. Antibiotic susceptibility was performed using the CLSI disk methods with Mueller-Hinton Media. Wound, skin and soft tissue specimens were incubated between 35–36°C in air. Respiratory specimens and subcultures from positive blood culture bottles were grown in CO_2_ enriched conditions using the candle jar method.

### Statistical analysis

Incidence was calculated using total population, age and sex data from the 2017 Fiji Bureau of Statistics census [15] and expressed per 100,000 census population per year with 95% confidence intervals (CI). As ethnicity data were unavailable from the 2017 census, denominators for ethnicity were calculated by applying Northern Division specific ethnicity proportions from 2007 census data to the 2017 population census data.[21] Non-overlapping CIs were interpreted as significant difference between the groups. We calculated incidence rate ratios (IRR) to compare population subgroups. We used Stata version 15 (StataCorp, College Station, Texas) for statistical calculations.

Patients diagnosed with more than one category of SSTI were assigned a principal diagnosis based on the most severe and most specific diagnosis, ranked according to a study-specific scale (S1 Fig). Principal diagnoses were categorized into two groups: 1) potentially scabies-related (cellulitis, abscess, impetigo, infected scabies, crusted scabies, pyomyositis necrotizing fasciitis with pure growth of *S*. *aureus* or GAS; and 2) unlikely scabies-related (wound infections, surgical wound infections and necrotizing fasciitis without pure growth of *S*. *aureus* or GAS). Sub-analysis for demographic groups was conducted for age, sex and ethnicity. Participant ethnicity was classified as either iTaukei or Other ethnicities (which included Fijians of Indian Descent and all other ethnicities). The outcomes that were assessed were: length of stay, need for amputation and survival.

## Results

### Incidence

During the 48-week study period there were 788 individual admissions of people with SSTIs who met inclusion criteria, corresponding to an annual incidence of 647.1 admissions per 100,000 people in the Northern Division (95% CI 602.9–693.8). Consent was obtained to collect more detailed demographic and clinical data for 748 admissions. Of these, 569 cases had principal diagnoses that were classified as potentially scabies-related (incidence of 467.3 per 100,000).

The overall annualized incidence of SSTI admissions was similar between males (684.2 per 100,000) and females (578.7 per 100,000 IRR 1.1, Table 1). Incidence was higher in the iTaukei population (752.7 per 100,000) compared to other ethnicities combined (442.2 per 100,000), with an IRR of 1.7 (95% CI 1.5–2.0, Table 1). The IRR between iTaukei and other ethnicities was similar in the potentially scabies-related (IRR 1.7) and unlikely scabies-related (IRR 1.8) groups.

**Table 1:** Hospital admissions for skin and soft tissue infections in Northern Division, by sex, ethnicity and age (Incidence is expressed as per 100,000 person-years, CI: confidence interval; IRR: incidence rate ratio, IRR between age groups was calculated using the 0–4 age group as reference).

Demographic factor		n	Incidence (95% CI)	IRR (95% CI)
**Sex**				
	Male	404	648.2 (586.7–714.4)	1.1 (1.0–1.3)
	Female	344	578.7 (519.3–643.0)	
**Ethnicity**				
	iTaukei	508	752.7 (688.9–820.8)	1.7 (1.5–2.0)
	Other	240	442.2 (338.1–501.7)	
**Age group (years)**				
	0–4	122	908.4 (754.9–1083.7)	
	5–14	89	351.6 (282.4–432.5)	0.4 (0.3–0.5)
	15–24	84	456.5 (364.3–564.9)	0.5 (0.4–0.7)
	25–34	94	559.1 (452–683.8)	0.6 (0.5–0.8)
	35–44	79	493.7 (391–615)	0.5 (0.4–0.7)
	45–54	99	696.2 (566.1–847)	0.8 (0.6–1.0)
	55–64	98	958.5 (778.8–1167)	1.1 (0.8–1.4)
	≥65	83	1127.3 (898.8–1395.8)	1.2 (0.9–1.7)
**Total**		**748**	**614.3 (571.2–659.8)**	

**Fig 2:**
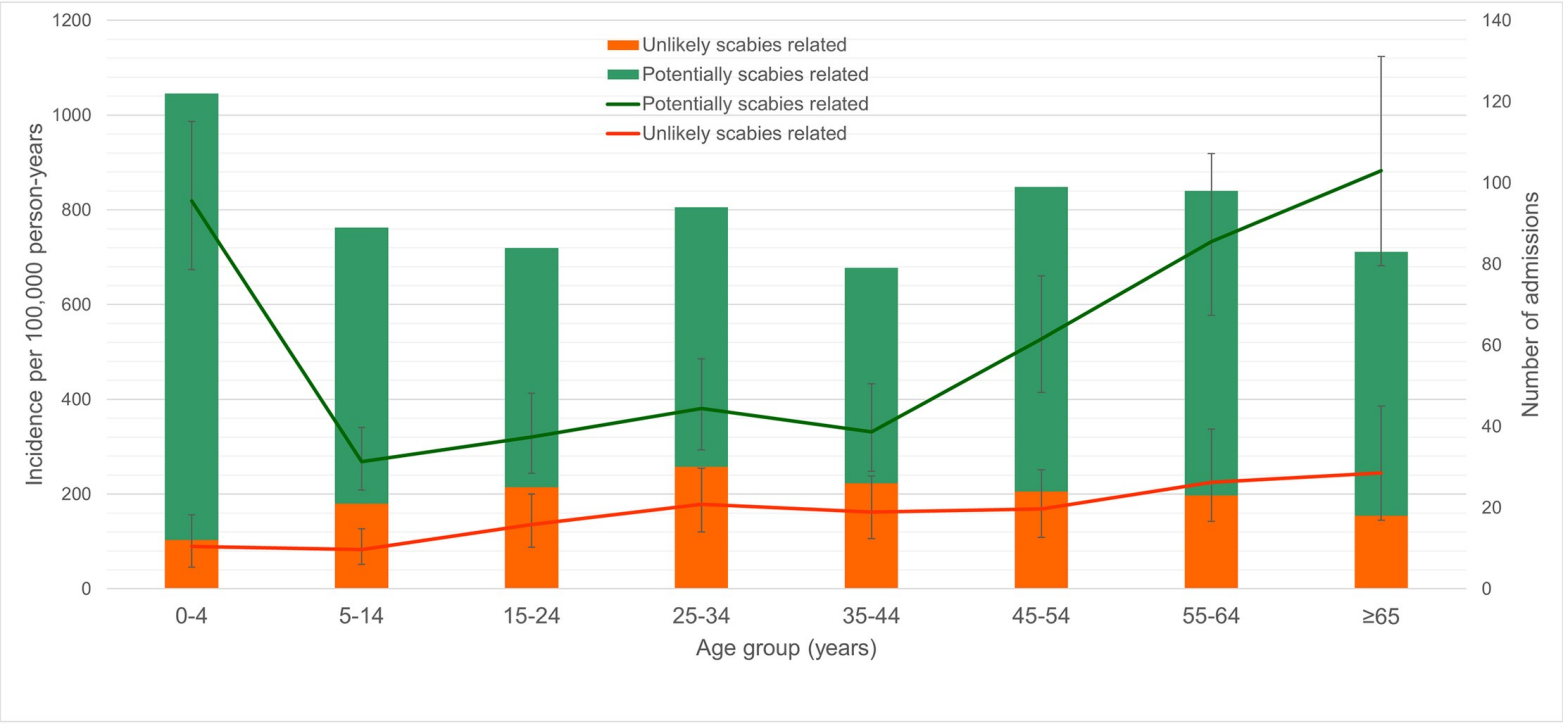
Admission numbers and age-specific incidence of patients admitted with a skin and soft tissue infection in the Northern Division of Fiji, July 2018 to June 2019. Data are differentiated into potentially scabies-related and unlikely scabies-related diagnoses. The number of cases for each age groups are shown by the columns; age-specific annual incidence is expressed per 100,000 population represented by the line graph, with 95% confidence intervals.

Incidence was highest in the youngest (<5 years) and oldest (55–64 years and ≥65 years) age groups, with incidence of 908, 958 and 1127 per 100,000 respectively (Table 1). The bimodal peaks in incidence among the youngest and oldest age groups were found in the group classified as potentially scabies-related but not in the unlikely scabies-related group (Fig 2).

A total of 556 admissions (74.3%) were among residents of Macuata Subdivision, where Labasa is located. Of these, 485 cases were direct admissions to Labasa Hospital, and 71 were referred from other health facilities. All-age incidence of admissions among residents of Macuata was 912.9 per 100,000 (95% CI 838.8–991.6). Based on the remaining 192 cases the combined incidence in the other three subdivisions was 315.5 per 100,000 (95% CI 272.5–363.3).

### Clinical characteristics

Of the 748 cases for which detailed clinical data were available, 52 (7%) had 2 SSTI diagnoses and 2 (0.3%) had 3. The most common principal diagnosis was abscess (398 cases, incidence 326.9 per 100,000) followed by cellulitis (106 cases, 87.1 per 100,000, Table 2).

**Table 2:** Incidence of admissions for skin and soft tissue infections by sex, ethnicity and broad age groups. (In the event that an individual case was diagnosed with more than one of the above conditions, a principal diagnosis was assigned using a study-specific scale outlined in S1 Fig. 95% CI: confidence interval).

	Abscess (N = 398)	Cellulitis (N = 106)	Surgical wound infection (N = 86)	Wound infection (N = 71)	Infected scabies (N = 27)	Pyomyositis (N = 27)	Necrotising fasciitis (N = 24)	Impetigo (N = 8)	Crusted scabies (N = 1)	All cases (N = 748)
**Gender**										
**Male**										
n	211	51	33	62	15	16	11	5	0	404
Incidence*	338.5	81.8	52.9	99.5	24.1	25.7	17.6	8.0	-	648.2
95% CI	294.5–387.4	60.9–107.6	36.4–74.4	76.3–127.5	13.5–39.7	14.7–41.7	8.8–31.6	2.6–18.7	-	586.7–714.4
**Female**										
n	187	55	53	9	12	11	13	3	1	344
Incidence*	314.6	92.5	89.2	15.1	20.2	18.5	21.9	5.0	1.7	578.7
95% CI	271.2–363.0	69.7–120.4	66.8–116.6	6.9–28.7	10.4–35.3	9.2–33.1	11.7–37.4	1.0–14.7	0.0–9.4	519.3–643.0
**Ethnicity**										
**iTaukei**										
n	261	66	57	52	25	24	16	6	1	508
Incidence*	386.7	97.8	84.5	77.1	37.0	35.6	23.7	8.9	1.5	752.7
95% CI	341.3–436.5	75.6–124.4	64.0–109.4	57.5–101.0	24.0–54.7	22.8–52.9	13.6–38.5	3.2–19.3	0.0–8.3	688.9–820.8
**Other**										
n	137	40	29	19	2	3	8	2	0	240
Incidence*	252.4	73.7	53.4	35.0	3.7	5.5	14.7	3.7	0.0	442.2
95% CI	212.0–298.3	52.7–100.3	35.8–76.7	21.1–54.7	0.4–13.3	1.1–16.2	6.4–29.0	0.4–13.3	0.0–6.8	388.1–501.7
**Age group**										
**0–14 years**										
n	110	25	5	26	22	13	2	7	1	211
Incidence*	283.9	64.5	12.9	67.1	56.8	33.6	5.2	18.1	2.6	544.6
95% CI	233.4–342.1	41.8–95.2	4.2–30.1	43.8–98.3	35.6–86	17.9–57.4	0.6–18.6	7.3–37.2	0.1–14.4	473.7–623
**15–54 years**										
n	199	36	65	30	3	13	10	0	0	356
Incidence*	304.1	55.0	99.3	45.8	4.6	19.9	15.3	-	-	544
95% CI	263.4–349.4	38.5–76.2	76.7–126.6	30.9–65.4	0.9–13.4	10.6–34	7.3–28.1	-	-	489.1–603.4
**≥55 years**										
n	89	45	16	15	2	1	12	1	0	181
Incidence*	506.1	255.9	91	85.3	11.4	5.7	68.2	5.7	-	1029.2
95% CI	406.6–622.4	186.7–342.2	(52–147.7)	47.7–140.6	1.4–41.1	0.1–31.7	35.3–119.2	0.1–31.7	-	885.3–1189.7
**Total**										
n	398	106	86	71	27	27	24	8	1	748
Incidence*	326.9	87.1	70.6	58.3	22.2	22.2	19.7	6.6	0.82	614.3
95% CI	295.6–360.5	71.3–105.3	56.5–87.2	44–75	13.8–33.3	13.8–33.3	12.2–30.9	2.8–14.4	0–5.6	571.2–659.8

The distribution of most common diagnoses was similar between males and females, with the exception of non-surgical wound infection which was far more common in males (99.5 per 100,000) compared to females (15.1 per 100,000) with an IRR of 6.8 (95% CI 3.2–15.0, Table 2). Median age varied between diagnoses (S2 Fig), most notably for infected scabies (1.6 years, IQR 0.8–6.4) and necrotizing fasciitis (56.3 years, IQR 44.8–60.8). iTaukei patients had a higher incidence of admission for infected scabies compared to other ethnicities combined (IRR 10.1, 95% CI 2.5–87.6, Table 2).

Fifty-seven patients (7.6%) were recorded as having scabies at the time of admission, including 7.6% of patients with cellulitis and 4.3% with skin abscesses. Scabies was most commonly recorded in younger children (24.6% of children aged < 5 years, S3 Fig). Scabies was more common among iTaukei (9.4%) patients compared to other ethnicities combined (2.5%, IRR 3.8, 95% CI 3.2–4.4). Scabies was diagnosed in 52 (9.1%) of potentially scabies-related SSTIs and 2 (1.1%) of other cases.

#### Management

Surgical intervention for SSTIs was performed in 478 patients (63.9%), including 87.5% of those with necrotizing fasciitis (Table 3). Eighteen patients (2.4%) required amputation of the affected extremity. Thirty-two patients (4.3%) were managed in the ICU (annual incidence 26.3 per 100,000), and 17 (2.3%) patients required mechanical ventilation.

**Table 3:** Treatment and outcome for patients admitted with a skin and soft tissue infection (CI: confidence interval; IQR: interquartile range; ICU: intensive care unit; One patient with crusted scabies is not represented in this table).

	Abscess (N = 398)	Cellulitis (N = 106)	Surgical wound infection (N = 86)	Wound infection (N = 71)	Infected scabies (N = 27)	Pyomyositis (N = 27)	Necrotizing fasciitis (N = 24)	Impetigo (N = 8)	All cases (N = 748)
**Surgery**									
n	316	18	46	53	1	22	21	1	478
%	79.4	17.0	53.5	74.7	3.7	81.5	87.5	12.5	63.9
95% CI	75.1–83.3	10.4–25.5	42.4–64.3	62.9–84.2	0.1–19	61.9–93.7	67.6–97.3	0.3–52.7	60.3–67.4
**ICU admission**									
n	11	1	3	4	7	0	3	3	32
%	2.8	0.9	3.5	5.6	25.9	0	12.5	37.5	4.2
95% CI	1.4–4.9	0–5.1	0.1–9.9	1.6–13.8	11.1–46.3	-	2.7–32.4	8.5–75.5	2.9–6
**IV antibiotics (days)**									
median	3	5	5	4	6	6	12.5	9.5	4
IQR	2–5	3–7	3–8	3–7	3–9	4–12	7–19	5–15	3–7
**Admission (days)**									
median	4	6	7	5	7	7	23	16.5	5
IQR	3–6	4–9	4–14	4–9	4–12	5–15	8–29.5	8–35	4–9
**Amputation**									
n	6	0	5	5	0	0	2	0	18
%	1.5	0	5.8	7.0	0	0	8.3	0	2.4
95% CI	0.6–3.3	-	1.9–13.1	2.3–15.7	-	-	1–27	-	1.4–3.8
**Died**									
n	6	5	2	6	0	0	5	1	25
%	1.5	4.7	2.3	8.5	0	0	20.8	12.5	3.3
95% CI	0.6–3.3	1.6–10.7	0.3–8.2	3.2–17.5	-	-	7.1–42.2	0.3–52.7	2.2–4.9

Treatment with intravenous antibiotics was commenced in 726 cases (97.1%), with a median duration of 4 days (IQR 3–7), longest for necrotizing fasciitis (12.5 days; IQR 7–19, Table 3). Cloxacillin was the most frequently prescribed intravenous antibiotic (90.1% of cases), followed by gentamicin (70%). Of all 518 cases that were prescribed gentamicin, 515 (99.4%) were treated in combination with another intravenous antibiotic, the majority being with cloxacillin (499, 96.3%). Oral antibiotic treatment was commenced in 550 cases (73.5%), with a median duration of 3 days (IQR 2–5). A total of 4007 and 2432 days of intravenous and oral antibiotics were prescribed over the 48-week surveillance period respectively.

#### Outcomes

The median length of stay in hospital was 8 days (IQR 4–9). Of all principal diagnoses, cases with abscess had the shortest stay (median 4 days, IQR 3–6) and necrotizing fasciitis the longest (23 days, IQR 8–29.5, Table 3). Over the 48-week surveillance period, admissions for SSTIs utilised a total of 5989 inpatient bed days and 150 ICU bed days.

Among the 748 patients for whom consent was obtained to provide clinical data, there were 25 deaths during admission (case fatality rate, CFR, 3.3%) corresponding to an annualised death rate of 20.5 per 100,000 (95% CI 13.3–30.3, S1 Table). After inclusion of a further 11 patients who died before consent for detailed clinical data could be sought, there were 32 deaths (CFR 4.1%; incidence 26.3 per 100,000, 95% CI 18.0–37.1).

Necrotizing fasciitis had the highest CFR (5 deaths, 20.8%, Table 3). The CFR for those aged <14 years was 0.5%, while the CFR for those aged over 65 years was 10.8% (incidence 122.2 per 100,000, 95% CI 55.9–231.9, S1 Table). The CFR was higher in patients with microbiologically confirmed bacteraemia (14.5% compared to those without 2.2%, IRR of 6.6, 95% CI 5.1–8.5). The CFR for patients admitted to the ICU was 12.5% compared to 2.9% (IRR 4.3, 95% CI 1.1–12.6) in cases without ICU admission.

### Microbiological findings

Superficial skin swabs were recorded as having been obtained from 520 patients (69.5% of consented cases). Of these, 409 (78.7%) yielded positive cultures, of which the majority were polymicrobial (221 swabs, 54%). Out of all swabs collected, *S*. *aureus* was isolated from 239 swabs (46%), including 11 (4.6% of all *S*. *aureus* isolated) that were methicillin-resistant, while GAS was isolated from 18 swabs (3.5%). Gram negative bacteria were isolated from 191 swabs (36.7%), most frequently *Klebsiella pneumoniae* (75 swabs, 14.4%, S2 Table).

Blood cultures were recorded as having been conducted on specimens from 431 patients (57.6%), with 64 returning positive (14.8%, S3 Table). *S*. *aureus* was isolated from blood cultures from 41 patients (9.5%), and GAS from 7 (1.6%). There was no significant variation with age in the incidence of SSTI admissions with *S*. *aureus* bacteraemia (S4 Table).

## Discussion

We observed an incidence of 647 SSTI admissions per 100,000. This rate is very high compared to the few other available reports from other parts of the world.[12–14] Patients admitted with a SSTI experienced a high CFR, up to 10.8% in those aged ≥65 years. Admissions with SSTI also imposed a substantial burden on the health care system with inpatient bed days for SSTIs over the surveillance period accounting for 10% of the hospital’s total bed capacity.

Incidence per capita was highest in young children and in the elderly, consistent with the age-prevalence relationship observed for both scabies and impetigo in Fiji[2] providing support for the hypothesis that these conditions are substantial contributors to SSTIs. This hypothesis is further supported by the finding in our study that SSTIs grouped as more likely to be scabies-related had a higher incidence in the young and the elderly, while those we grouped as unlikely to be scabies-related were evenly distributed across all ages. There may be other factors that explain this distribution however, such as higher GAS pharyngeal carriage in children resulting in increased bacterial transmission, [22,23] or predisposing comorbidities such as Type 2 diabetes mellitus in older age groups.[24]

While our finding of a very low proportion of *S*. *aureus* positive skin swabs being methicillin resistant (4.6%) is consistent with previous studies in Fiji, [25] this is a much lower proportion compared to other populations such as the United States (46%), Latin America (29.4%), Europe (22.8%) and China (20%).[11,26,27] The proportion of skin swabs that were positive for gram-negative bacteria is consistent with that described in other island populations with similar proportions of *Klebsiella pneumoniae*.[28]

The overall CFR of 4.3%, was substantially higher than that reported in high- and other middle-income settings; studies in the US and China have reported CFRs of 0.4% and 2.4% respectively [26,29]. The reason for this difference is unclear; one possible explanation is late admission, supported by the finding that the majority (63.9%) of patients underwent surgery, compared to 44% and 37.1% in the US and China respectively.[26,29] We observed that patients in our study required a median length of stay of 8 days, 97% required intravenous antibiotics, 63.9% surgery and 4.2% required admission to the ICU (incidence 26.3 per 100,000), reflecting the severity of disease presentations.[26,30]

Comprehensive skin health measures including health promotion, environmental optimisation, dissemination of treatment guidelines and even screening can be moderately effective in reducing prevalence of scabies and impetigo,[31] but there are no studies of the impact of these interventions on more complicated SSTIs. Ivermectin-based MDA for scabies control has been shown to substantially reduce scabies and impetigo prevalence in Pacific Island communities.[4,32] However, there is no current information on whether this leads to prevention of more complicated bacterial SSTIs or invasive infections. We collected these data in the context of a large before-after intervention trial, delivering two doses of ivermectin-based MDA to the entire Northern Division of Fiji. The trial aims to determine the impact of ivermectin-based MDA on infectious complications of scabies, in particular, bacterial SSTIs.[4,32]

There are a number of limitations to our study. First, it is possible that the true incidence of scabies in our recruited cases was underestimated, because of “normalisation” of scabies by clinicians in endemic settings where scabies is very common.[33] The reliance on hospital procedures for assessment and diagnosis may have led to under-reporting of cases admitted to hospital with an SSTI. Second, the incidence of SSTI admissions we report here for the Northern Division is a lower bound to the true number of SSTI cases that require hospitalization, because some SSTIs requiring hospitalization but lower levels of surgical or medical intervention are managed at the three small subdivisional hospitals. When we restricted our analysis to residents of Macuata and used the population of Macuata as the denominator, the incidence of SSTI admissions from within Macuata was three times higher than the combined incidence from the other subdivisions.

Our study highlights the very high disease burden of SSTIs in Fiji, especially among the extremes of age and iTaukei population. It is likely that there is a similarly high burden of SSTIs in other tropical scabies-endemic countries, including those in the Pacific region, and further studies are needed to better understand the epidemiology and health impact of SSTIs in these settings. Our findings highlight the need for investigation into strategies to reduce the incidence and impact of SSTIs in Fiji and in other tropical countries where SSTIs are common, including MDA for scabies.

## Supporting information

S1 Fig Selection approach for principal diagnosis. (TIF)

S2 Fig Median age at admission by condition. The line within the boxplot indicates the median age at admission in years, the upper and lower borders of the box represent the interquartile range, the error bars represent minimum and maximum values and dots represent outliers.
(TIF)

S3 Fig Percentage of skin and soft tissue admissions diagnosed with scabies by age group. The columns demonstrate the percentage of patients admitted with skin and soft tissue infection that were also diagnosed with scabies.
(TIF)

S1 Table Case fatality rate and incidence of death by age group. (PDF)

S2 Table Breakdown of positive culture results for skin swabs. (PDF)

S3 Table Breakdown of positive blood culture results. (PDF)

S4 Table Incidence of Staphylococcus aureus bacteraemia among admissions with skin and soft tissue infections by age group. (PDF)

S1 Data Full data set. (XLSX)
