# Supplementary figures and images for: Hospital admissions for skin and soft tissue infections in a population with endemic scabies: A prospective study in Fiji, 2018–2019
Source: PLoS Negl Trop Dis. 2020 Dec 9;14(12):e0008887. doi: 10.1371/journal.pntd.0008887 (PMC7752096; doi:10.1371/journal.pntd.0008887)

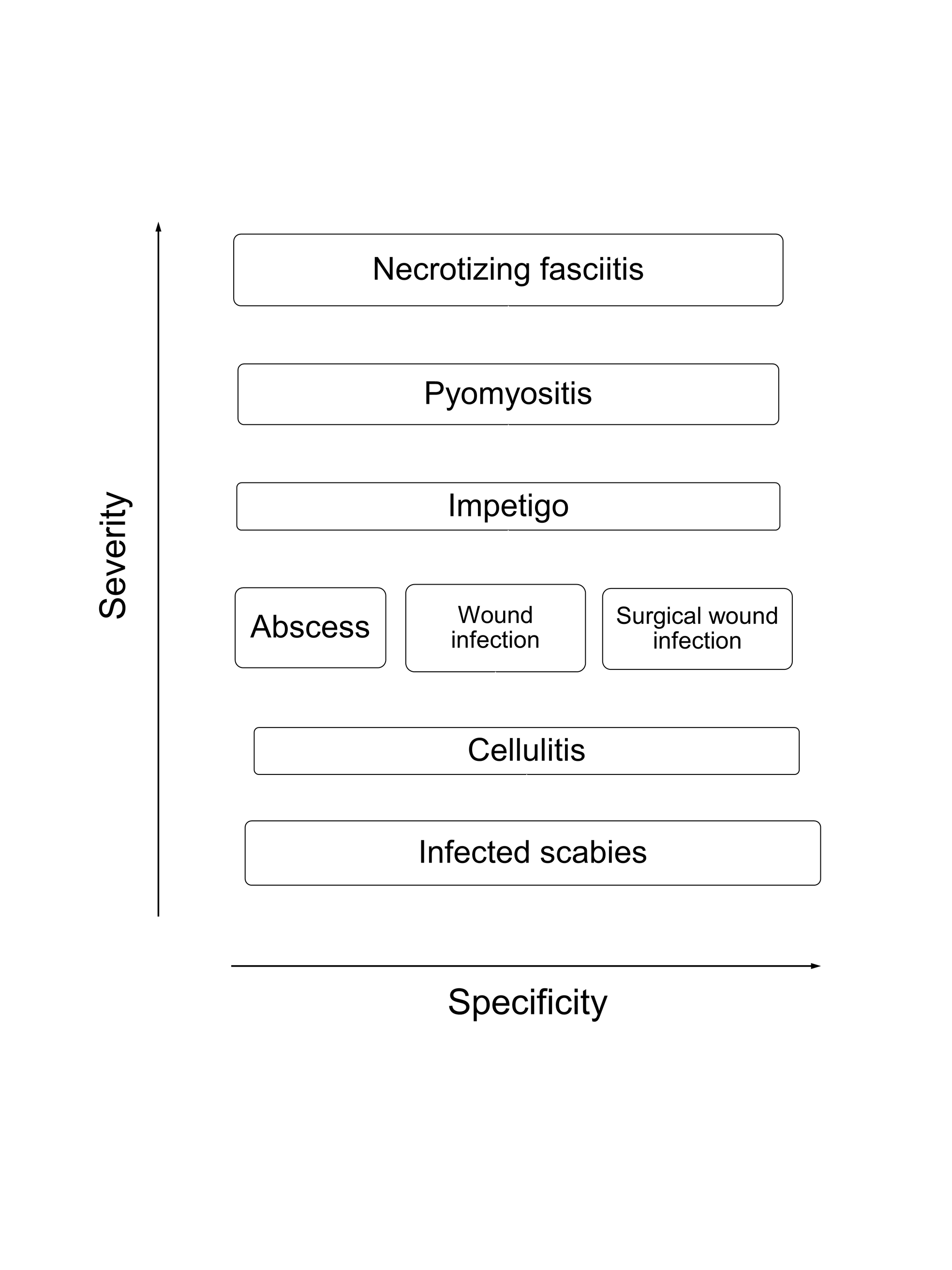

Supplement: S1 Fig — (TIF) [file pntd.0008887.s001.tif]

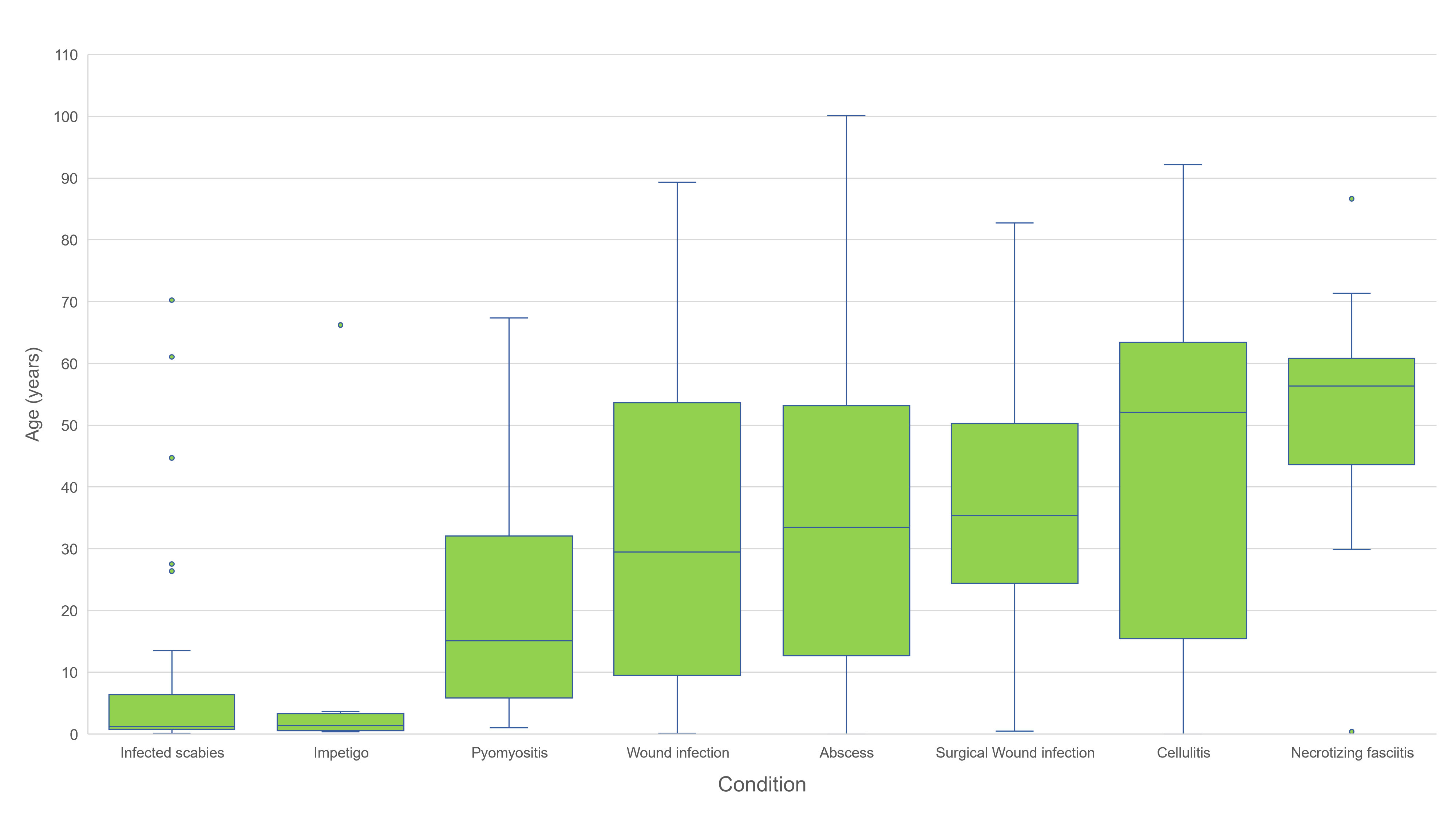

Supplement: S2 Fig — The line within the boxplot indicates the median age at admission in years, the upper and lower borders of the box represent the interquartile range, the error bars represent minimum and maximum values and dots represent outliers. (TIF) [file pntd.0008887.s002.tif]

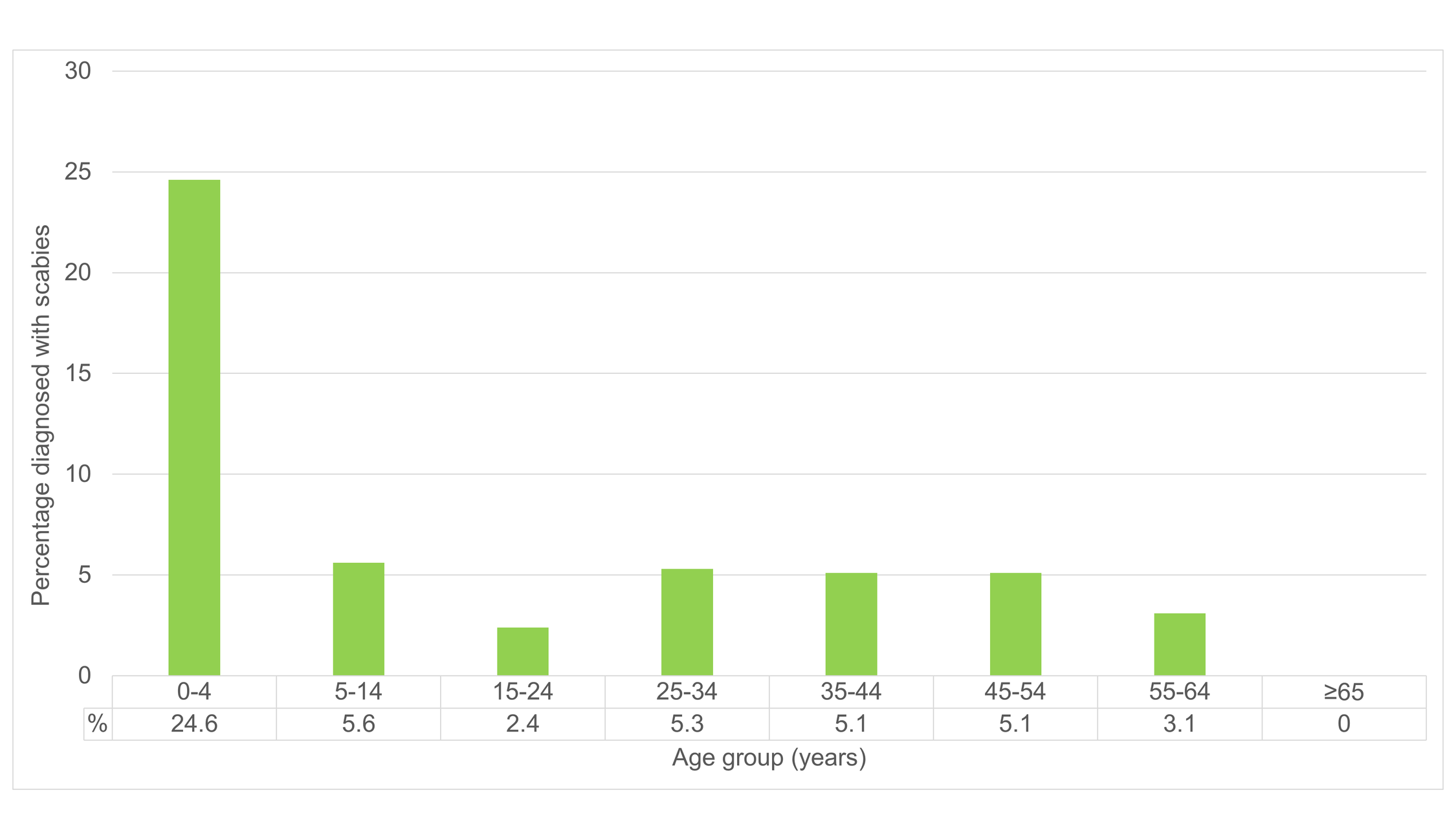

Supplement: S3 Fig — The columns demonstrate the percentage of patients admitted with skin and soft tissue infection that were also diagnosed with scabies. (TIF) [file pntd.0008887.s003.tif]
