# Supplementary material for: Hospital admissions for skin and soft tissue infections in a population with endemic scabies: A prospective study in Fiji, 2018–2019
Source: PLoS Negl Trop Dis. 2020 Dec 9;14(12):e0008887. doi: 10.1371/journal.pntd.0008887 (PMC7752096; doi:10.1371/journal.pntd.0008887)
Supplement: S1 Table — (PDF) [file pntd.0008887.s004.pdf]

| Age group (years) | Number of deaths | Total population | Number admitted | Case fatality rate % (95%CI) | Annual incidence/100,000 (95%CI) |
|---|---|---|---|---|---|
| 0-4 | 1 | 14550 | 122 | 0.8 (0.2-4.5) | 7.4 (0.1-41.5) |
| 5-14 | 0 | 27423 | 89 | 0 | 0 |
| 15-24 | 2 | 19934 | 84 | 2.4 (0.3-8.3) | 10.9 (1.3-39.3) |
| 25-34 | 1 | 18214 | 94 | 1.1 (0-5.8) | 5.9 (0.2-33.1) |
| 35-44 | 4 | 17335 | 79 | 5.1 (1.4-12.5) | 25.0 (6.8-64) |
| 45-54 | 2 | 15406 | 99 | 2.0 (0.2-7.1) | 14.1 (1.7-50.8) |
| 55-64 | 6 | 11076 | 98 | 6.1 (2.3-12.9) | 58.7 (21.5-127.7) |
| >=65 | 9 | 7976 | 83 | 10.8 (5.1-19.6) | 122.2 (55.9-231.9) |
