# Supplementary material for: Hospital admissions for skin and soft tissue infections in a population with endemic scabies: A prospective study in Fiji, 2018–2019
Source: PLoS Negl Trop Dis. 2020 Dec 9;14(12):e0008887. doi: 10.1371/journal.pntd.0008887 (PMC7752096; doi:10.1371/journal.pntd.0008887)
Supplement: S2 Table — (PDF) [file pntd.0008887.s005.pdf]

**Bacteria**	**Number of cases**	**% of all skin swabs (N=520)**	**% of culture-positive swabs (N=409)**
Staphylococcus aureus			
total	239	46	58.4
methicillin sensitive	228	43.8	55.7
methicillin resistant	11	2.1	2.7
Klebsiella pneumoniae	75	14.4	18.3
Pseudomonas aeruginosa	38	7.3	9.3
Enterobacter spp.	34	6.5	8.3
Escherichia coli	32	6.2	7.8
Proteus mirabilis	26	5	6.4
Acinetobacter baumannii	20	3.9	4.9
GAS	18	3.5	4.4
Coagulase negative *Staphylococcus*	11	2.1	2.7
Strep sp. (unspecified)	10	1.9	2.4
Morganella morganii	6	1.2	1.5
Acinetobacter spp.	5	1	1.2
Enterococcus spp.	4	0.8	1
Klebsiella oxytoca	4	0.8	1
Group B Streptococcus	4	0.8	1
Citrobacter diversus	4	0.8	1
Group G Streptococcus	3	0.6	0.7
Serratia marcescens	3	0.6	0.7
Pseudomonas fluorescens	2	0.4	0.5
Group C Streptococcus	1	0.2	0.2
Providencia spp.	1	0.2	0.2
Bacillus spp.	1	0.2	0.2
Candida spp.	1	0.2	0.2
