# Supplementary material for: Hospital admissions for skin and soft tissue infections in a population with endemic scabies: A prospective study in Fiji, 2018–2019
Source: PLoS Negl Trop Dis. 2020 Dec 9;14(12):e0008887. doi: 10.1371/journal.pntd.0008887 (PMC7752096; doi:10.1371/journal.pntd.0008887)
Supplement: S3 Table — (PDF) [file pntd.0008887.s006.pdf]

**Organism cultured**	**Number of cases**	**% of all blood cultures taken (N=423)**	**% of positive cultures (N=64)**
Staphylococcus aureus			
Total	41	9.7	64.1
methicillin sensitive	40	9.5	62.5
methicillin resistant	1	0.2	1.6
GAS	7	1.7	10.9
Klebsiella pneumoniae	5	1.2	7.8
Pseudomonas aeruginosa	3	0.7	4.6
Escherichia coli	3	0.7	4.6
Staphylococcus saprophyticus	3	0.7	4.6
Streptococcus pneumoniae	1	0.2	1.6
Enterobacter cloacae	1	0.2	1.6
Klebsiella (Enterobacter) aerogenes	1	0.2	1.6
Aeromonas hydrophila	1	0.2	1.6
HACEK group (unspecified)	1	0.2	1.6
Candida tropicalis	1	0.2	1.6
