# Supplementary material for: Hospital admissions for skin and soft tissue infections in a population with endemic scabies: A prospective study in Fiji, 2018–2019
Source: PLoS Negl Trop Dis. 2020 Dec 9;14(12):e0008887. doi: 10.1371/journal.pntd.0008887 (PMC7752096; doi:10.1371/journal.pntd.0008887)
Supplement: S4 Table — (PDF) [file pntd.0008887.s007.pdf]

**Age group (years)**	***Staphylococcus aureus* bacteraemia**	**Total population**	**Annual incidence**	**95% confidence intervals**
0-4	12	14550	89.3	46.2-156
5-14	9	27423	35.6	16.3-67.5
15-24	9	19934	48.9	22.4-92.8
25-34	7	18214	41.6	16.7-85.8
35-44	6	17335	37.5	13.8-81.6
45-54	11	15406	77.4	38.6-138.4
55-64	7	11076	68.5	27.5-141
>=65	8	7976	108.7	46.9-214
